# The use of HPLC-Q-TOF-MS for comprehensive screening of drugs and psychoactive substances in hair samples and several “legal highs” products

**DOI:** 10.1007/s00706-016-1773-z

**Published:** 2016-06-07

**Authors:** Justyna Aszyk, Agata Kot-Wasik

**Affiliations:** Department of Analytical Chemistry, Faculty of Chemistry, Gdańsk University of Technology, Gdańsk, Poland

**Keywords:** Drug research, Mass spectroscopy, Extraction, Quadrupole time-of-flight mass analyzer

## Abstract

**Abstract:**

Non-targeted screening of drugs present in herbal products, known as “legal high” drugs and in hair as a biological matrix commonly used in toxicological investigations was accomplished with the use of high pressure liquid chromatography coupled with quadrupole time-of-flight mass spectrometry (HPLC-Q-TOF-MS). In total, 25 and 14 therapeutical drugs and psychoactive substances/metabolites were detected in investigated hair samples and herbal products, respectively. We demonstrate that the HPLC-Q-TOF methodology seems to be a powerful tool in the qualitative analysis applied in identification of these designer drugs, thus enabling a laboratory to stay-up-to-date with the drugs that are being sold as legal high products on black market.

**Graphical abstract:**

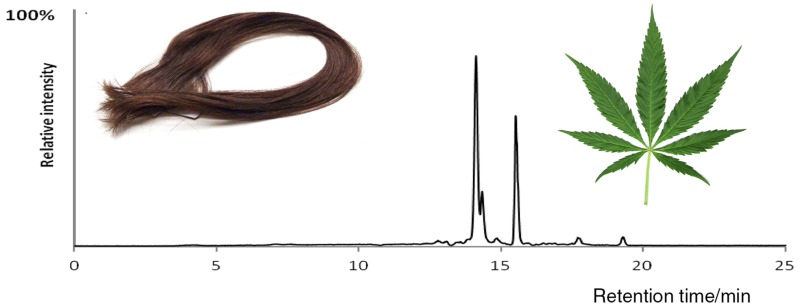

## Introduction

The identification of large number of new continuously and increasingly appearing designer drugs on drug market is currently a major priority for forensic laboratories [1, 2]. Due to the novelty of incoming psychoactive products known as “legal highs” or “herbal highs”, at present there is limited research in the published literature concerning the pharmacokinetics, pharmacological or toxicological effects of these drugs [3]. Control of production and distribution of new emerging legal highs is strongly dependent on implementation of new screening methods and analytical solutions. The identification of psychoactive substances is challenging due to their fast transience on the drug scene.

Recently, many analytical techniques have been applied in comprehensive drug screening in biological and non-biological specimens. Application of routine toxicological methods based on the use of immunochemical assays is limited mainly due to their insufficient sensitivity and limited coverage. Their use is also hampered by the risk of obtaining false positive results, what can lead to serious medical and social consequences [4, 5]. Therefore, high performance liquid chromatography coupled with high resolution TOF (or Q-TOF) mass spectrometry is mostly applied. HPLC-Q-TOF-MS technique enables tentative identification of unknown compound based on prediction of chemical formula from accurate ion mass measurement and characteristic isotopic pattern [6]. Moreover, the volume of sample required for analysis is very small [7]. Therefore, it is stated as a very powerful tool for identification of species and can be very useful when reference standards are not available. Moreover, it provides excellent full-scan sensitivity, that makes it suitable for wide-scope screening in forensic investigations. The successful application of Q-TOF-MS technique in screening of many different families of drugs has been reported by many researchers [5, 6, 8–10].

The availability of the legal high products on the black market, in various forms of preparations like powders, pills, and teas is increasing tremendously. These products are easily available in herbal shops. Moreover, they are intaken as an alternative for federally illegal amphetamines or opioids [4, 11].

In the last decades, hair analysis has become a well established strategy to investigate retrospectively drug abuse histories [4]. Hair, as a human matrix, exhibit a lot of highlights in drug of abuse analysis compared to other biological samples (blood or urine) [12]. Firstly, sampling step for hair is non-invasive, simple, and painless for the patient. Secondly, hair sample does not require any special storage and transport conditions due to slow process of hair destruction in comparison to another biological samples [13]. Besides, drugs can stay in this matrix for a long time (even months). However, hair samples have got some limitations for analysts, just to mention time-consuming analytical procedures and high correlation to melanin concentration dramatically affecting results [14]. In short, what is the most important, hair allows to retrospective detection of chronic exposure to drugs or poisons up to years back. Hair analysis consists of few principal steps: sampling, storage and transport, decontamination, extraction of features from biological matrix, instrumental analysis, and finally data interpretation. The decontamination phase involves of one or washings of the sample to eliminate possible external contamination. The extraction of the analytes from the hair can be achieved by various methods, which differ according to the nature of the analytes themselves and the identification technique to be employed [15].

The purpose of this paper was to investigate the capability of high pressure liquid chromatography coupled with quadrupole time-of-flight mass spectrometry for rapid screening of representative multiclass drugs including antidepressants, non-narcotic, antibiotics, or illegal drugs such as opioids and amphetamines in herbal products and hair samples taken as the one of the most commonly used as biological matrices. The high sensitivity obtained in full-scan MS mode allows to the retrospective detection of unknown compounds. Our aim was to present the overall concept of application of Q-TOF technique in two areas of toxicological screening: firstly, detection of chronic intake of therapeutical drugs and illegal substances in 13 hair samples obtained from volunteers, former addicts and secondly to evaluate the presence of federally controlled active substances in four commercially illegal highs products investigated under this study.

## Results and discussion

**Fig. 1:**
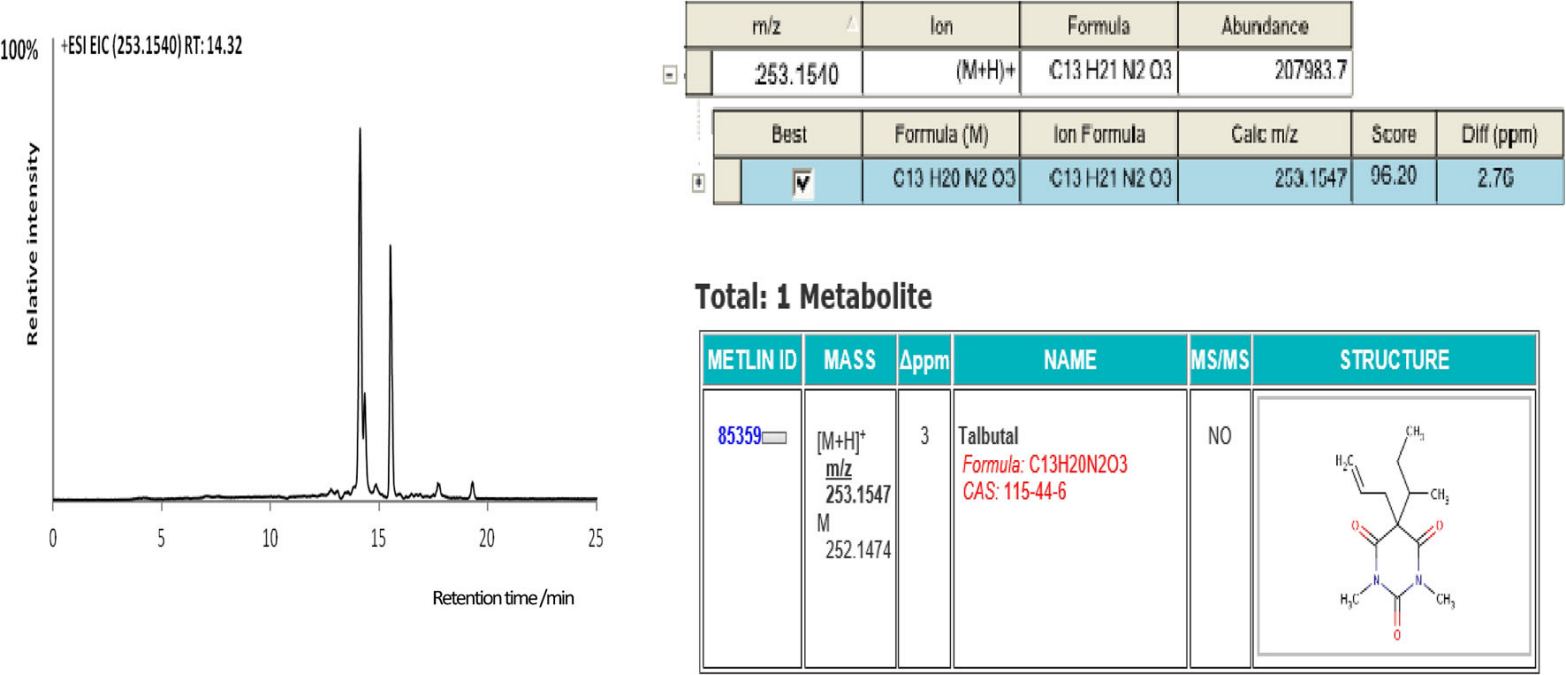
Steps in the identification of unknown compound with the use of METLIN database. Example was presented for talbutal detected in herbal product

**Fig. 2:**
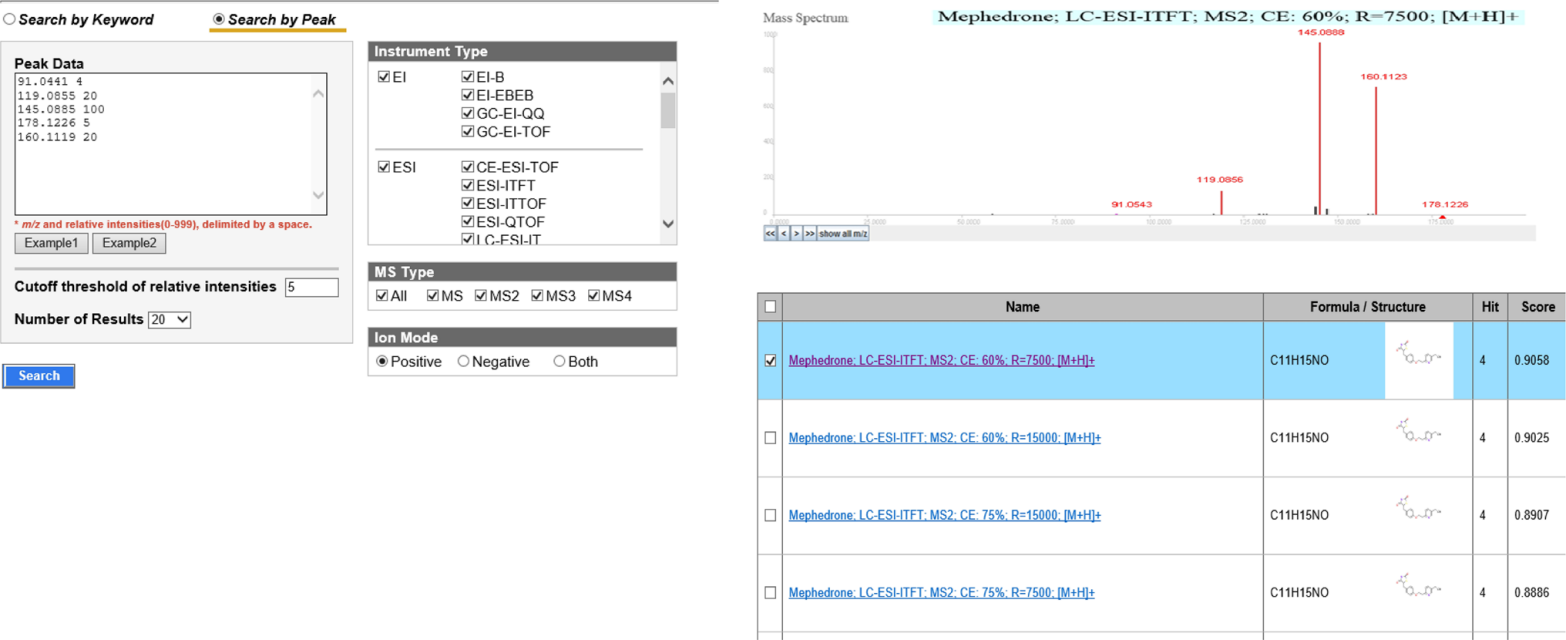
Steps in the identification of unknown compound with the use of MassBank database. Example was presented for mephedrone detected in herbal product

The automatic screening and identification of drugs present in hair and herbal products were based on the use of narrow mass window (±5 ppm) for (pseudo)molecular ion and utilization of isotope fit. Justification of the accurate masses of unknown compound fragments generated in MS tandem mode was additionally supported by its chemical structure and comparison of data previously reported in the literature. Additionally, identification of particular drug was facilitated by the use of commercially available NIST 2.0 Software with in-house database containing 885 compounds of forensic relevance. In case of failure searching of in-house personal compound database, screening of unknown species was accomplished with use of on-line databases, such as: METLIN, Mass Bank, ChemSpider, and PubChem. The overall workflow for the identification of compounds with use of METLIN’s Metabolite spectral database and MassBank database is presented in Figs. 1, 2, respectively.

**Fig. 3:**
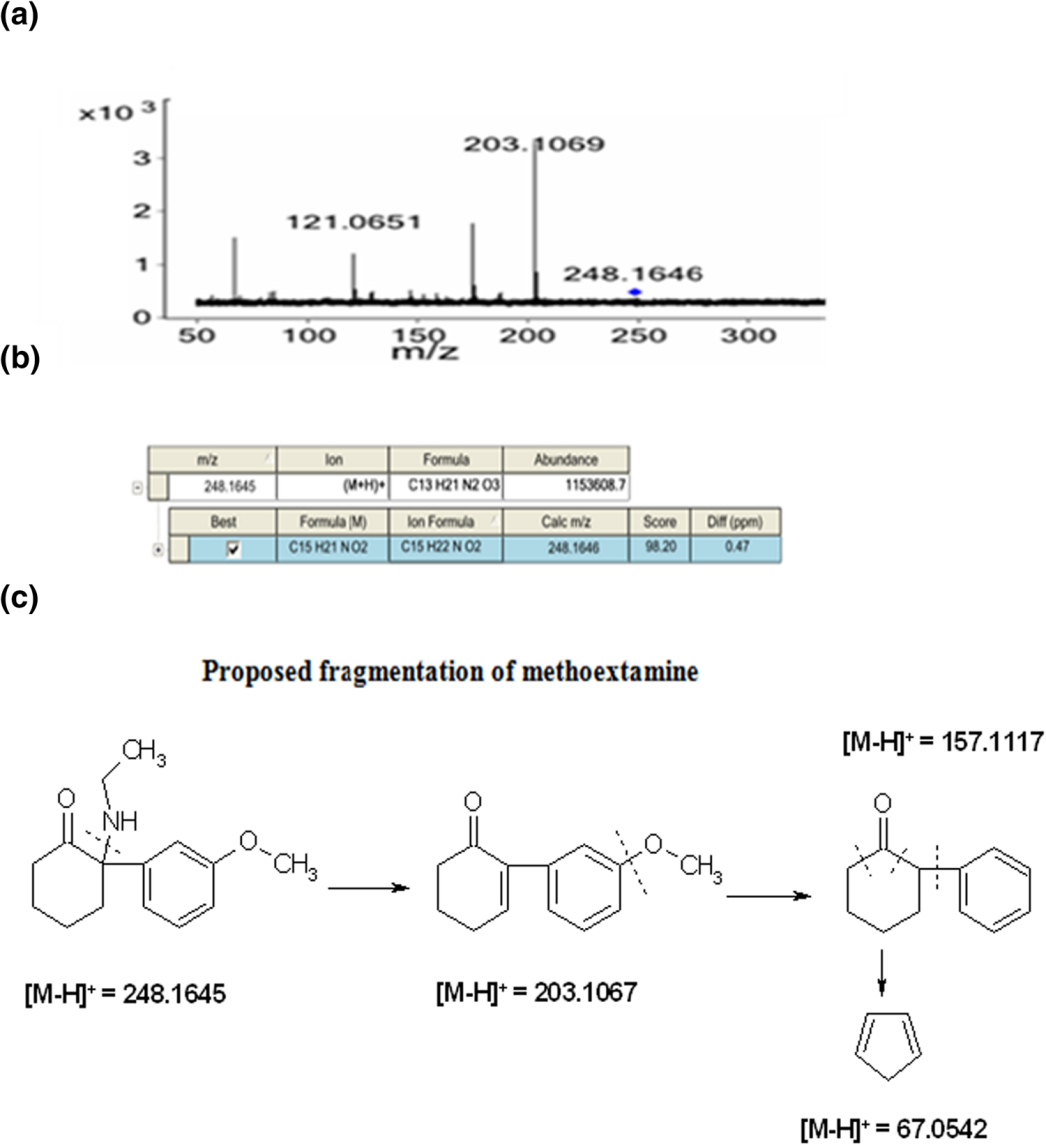
Steps in the identification of methoxetamine in hair samples by HPLC-Q-TOF-MS/MS: **a** MS/MS spectrum of ion at *m/z* = 248.1646; **b** generated chemical formula at *m/z* = 248.1646 –(C_15_H_21_NO_2_) with software MassHunter Workstation QualitativeAnalysis. B.03.01, measured mass: *m/z* = 248.1646; calculated mass [M+H]: 248.1645; **c** proposed fragmentation of methoxetamine

Identification of compounds based only on accurate mass measurement can lead to false positive results (in case of isobaric compounds); therefore, Target MS/MS mode was necessarily performed to obtain the fragmentation spectra of selected compounds and elucidate their possible structures. The overall concept of elucidation the structure of compound based on MS/MS spectrum was present using identification of methoxetamine in hair samples as an example (Fig. 3). Elemental composition analysis within the set criteria with use of MassHunter software generated a compound with *m/z* = 248.1646 with chemical formula C_15_H_21_NO_2_. The elemental composition indicates that this compound might be ethylphenidate or methoxetamine. The fragmentation of the particular compound by MS/MS provided four main fragment ions at *m/z* = 203.1069, 175.1116, 121.0651, and 67.0541. Observed fragments and comparison of supporting information from databases confirmed the fact that methoxetamine is predicted compound. It strongly reduces the number of unknown compounds to a single structure. A summary of the psychoactive substance detected in dried plant material and hair samples is shown in Tables 1, 2, respectively. In total, the inspection of the accurate mass MS total ion chromatogram (TIC) indicate the presence of 14 compounds in investigated legal high products and 25 in hair samples, respectively.

**Table 1:** Summary of drug detected in herbal products and their theoretical and experimental masses

Product name	Compounds detected	Chemical formula	*m/z* value		Δ*m*/ppm
			Measured	Calculated	
Tajfun	Mephedrone	C_11_H_15_NO	178.1224	178.1226	1.12
	Talbutal	C_13_H_20_N_2_O_3_	253.1540	253.1547	2.76
	JWH-016	C_24_H_23_NO	343.2767	343.2780	3.46
	JWH-369	C_26_H_24_ClNO	401.1867	401.1546	1.74
The recidivist	Ropinirole	C_16_H_24_N_2_O	261.1965	261.1961	1.53
	SB-206553	C_17_H_16_N_4_O	293.1814	293.1808	2.05
	UR-144	C_21_H_29_NO	312.2327	312.2322	1.60
	Cocaethylene	C_18_H_23_NO_4_	318.1689	318.1700	3.46
	Perazine	C_20_H_25_N_3_S	340.1846	340.1842	1.18
R.I.P.	Ethylketocyclazocine	C_19_H_25_NO_2_	300.1966	300.1958	2.66
	UR-144	C_21_H_29_NO	312.2312	312.2322	3.20
	Perazine	C_20_H_25_N_3_S	340.1847	340.1842	1.47
	AKB-48	C_23_H_31_N_3_O	366.2553	366.2540	3.55
The Bandit	Clorgiline	C_13_H_15_Cl_2_NO	272.0598	272.0603	1.84
	Cocaethylene	C_18_H_23_NO_4_	318.1690	318.1700	3.14
	Propoxyphene	C_22_H_29_NO_2_	340.2284	340.2271	3.82

**Table 2:** Summary of drug detected in hair samples and their theoretical and experimental masses

Compounds detected	Chemical formula	*m/z* value		Δ*m*/ppm
		Measured	Calculated	
6-APB	C_11_H_13_NO	176.1069	176.1070	0.57
6-APDB	C_11_H_15_NO	178.1230	178.1226	2.25
Amphetamine	C_9_H_13_N	136.1124	136.1121	2.20
Dextromethorphan	C_18_H_25_NO	272.2009	272.1998	4.04
Dimethyltryptamine	C_12_H_16_N_2_	189.1382	189.1386	2.11
Doxepin	C_19_H_21_NO	280.1692	280.1696	1.43
Desmethyldoxepin	C_18_H_19_NO	266.1542	266.1539	1.13
Hydroxydoxepin	C_19_H_21_NO_2_	296.1652	296.1645	2.36
Fentanyl	C_22_H_28_N_2_O	337.2267	337.2274	2.08
Fluoxetine	C_17_H_18_F_3_NO	310.1415	310.1413	0.64
Hydroxyzine	C_21_H_27_ClN_2_O_2_	375.1830	375.1834	1.07
Methadone	C_21_H_27_NO	310.2170	310.2165	1.61
Methoxetamine	C_15_H_21_NO_2_	248.1646	248.1645	0.40
α-Methyltryptamine	C_11_H_14_N_2_	175.1231	175.1230	0.57
Metropolol	C_15_H_25_NO_3_	268.1899	268.1907	2.98
Noopept	C_17_H_22_N_2_O_4_	319.1656	319.1652	1.25
Paracetamol	C_8_H_9_NO_2_	152.0704	152.0706	1.32
Perazine	C_20_H_25_N_3_S	340.1833	340.1842	2.65
Sulfamethoxazole	C_10_H_11_N_3_O_3_S	254.0590	254.0594	1.57
α-Tramadol	C_16_H_25_NO_2_	264.1951	264.1958	2.65
*O*-Desmethylotramadol	C_15_H_23_NO_2_	250.1799	250.1801	0.80
*N*-Desmethylotramadol	C_15_H_23_NO_2_	250.1799	250.1801	0.80
Trimethoprim	C_14_H_18_N_4_O_3_	291.1447	291.1452	1.72
Zopiclone	C_17_H_17_ClN_6_O_3_	389.1120	389.1123	0.77
UR-144	C_21_H_29_NO	312.2310	312.2322	3.84

The Controlled Substances Act (CSA) regulates import, possession, use, and distribution of certain substances. The legislation includes five schedules, that are describing the characteristics of each substance. Talbutal, as intermediate-acting barbiturate, belongs to substances controlled in Schedule III, which use can lead to moderate and psychical dependence. Synthetic cannabinoid compounds, such as UR-144, AKB-48, JWH-016, JWH 369 and synthetic stimulants, like mephedrone have been controlled by United States Drug Enforcement Agency under Schedule I (drugs with high potential of abuse) [16]. As can be seen in Table 1 federally controlled compounds were detected in commercially and easily available legal herbal products. After legislation and registration processes the use of these controlled substances is unexpectedly not waned, what confirms and enforces of the necessity of qualitative identification of this type of products in order to enable forensic laboratories to be “on time” with current drugs and its possible replacements being sold as designer products.

In the 13 hair samples, 21 therapeutic and illegal drugs were identified. This included mainly: sedative hypnotic drugs (zopiclone), antitussives (dextromethorphan, dimethyltryptamine), antidepressants (fluoxetine, doxepin), antihistaminics (hydroxyzine), non-narcotic analgesics (methadone, fentanyl, paracetamol), antibiotics (sulfamethoxazole, trimethoprim), antipsychotics (perazine), adulterants (hydroxyperazine), narcotic analgesics (α-tramadol), medications for treatment of cardiovascular diseases (metropolol), nootropics (noopept), stimulants and psychedelics (6-APB, 6-APBD, α-methyltryptamine) and illegal drugs such as opioids (methadone), amphetamines (amphetamine), cannabinoids (UR-144), cathinones (methoxetamine). The results are summarized in Table 2.

Additionally, metabolites of tramadol, namely *O*-desmethyltramadol and *N*-desmethyltramadol as well as metabolites of doxepin- hydroxydoxepin and desmethyldoxepin were found in hair samples. Due to similarity in doxepin structure (*m/z* = 280.1696) and desmethyldoxepin (*m/z* = 266.1539) these analytes were eluted at the same time. The intensity of hydroxydoxepin peak is higher than the intensity of doxepin peak, what can be a proof for higher incorporation of this metabolite in hair structure than other features. Despite the large advantages of use of HPLC-Q-TOF-MS technique for the screening of drugs in hair, there were 22 drugs and psychoactive substances mentioned in volunteer’s questionnaires but were not detected in the hair samples. The details were summarized in Table 3.

**Table 3:** Summary of analyzed hair samples with undetected substances

Subject no. X	Color of hair	Undetected substances	Additional information
III	Gray	Tolterodine	Washing hair at least one time per day
VIII	Dark blonde	Morphine	−
IX	Dark blonde	AM-2201, codeine, ephedrine, 4-AcO-DMT, methandienone, GBL, 2C-B, dimenhydrinate, 25C-NBOMe, 4-FMA, 5-Meo-MiPT	Not cutting hair for 5 years
XIII	Middle blonde	Ergine, 5-API, ethylphenidate, 2C-P, MAM-2201, 25C-NBOMe, MDMA, 25I-NBOMe, 5-HO-DMT	−

Few reasons can be given to explain this phenomena. Firstly, low incorporation rate of drugs in hair, which can be affected by washing-out by shampooing and hair pigmentation (sample III). It is well known, that the incorporation of drugs in the hair depends on melanin content in the matrix and is regulated by the pharmacological principles of the substance distribution. The incorporation and binding of drugs in the hair are much greater in pigmented versus non-pigmented hair, so no detection of these drugs in gray hair is explicable [13]. The reason can also lie in irregular intake of drugs (sample IX), insufficient stability of features in hair, a long-term medical treatment in case of some drugs and finally low concentration of drug in hair sample which is not sufficient for Q-TOF-MS detection (sample VIII). In case of sample IX, hair were collected from tip (distal) section of hair as well. This additional analysis was performed in order to verify how cutting/not-cutting of hair for 5 years (as was declared in questionnaire) will affect results. This effort allowed to detect 6-APB (this drug was not detected in hair sample taken from posterior vertex of the head), what confirms hypothesis that this drug was intaken in earlier period of life.

## Conclusions

In this study, a HPLC method coupled with Q-TOF-MS for the toxicological screening and identification of 39 drugs and metabolites in herbal products and hair samples was developed. The proposed HPLC-Q-TOF method based both on accurate mass, isotopic pattern recognition and fragmentation spectra obtained in Targeted MS/MS mode has been successfully applied to hair samples from 13 abusers with known therapeutic and psychoactive drug intake at the life time and 4 herbal legal high products. Positive and negative ion mode was applied in order to increase sensitivity for basic and acidic analytes. The sample preparation including basic incubation followed by liquid–liquid extraction with ethyl acetate was suitable for variety of substances present in investigated hair samples. Despite the large advantages of HPLC-Q-TOF-MS technique in comprehensive forensic investigations, a disagreement between substances mentioned in questionnaires and detected in hair in some cases was observed.

The developed HPLC-Q-TOF-MS method provides to be applicable in comprehensive forensic investigations, depending on the aim of the research: (1) introducing a legislation and restriction according to new federally uncontrolled substances detected in so called “legal highs”, (2) studies on mechanism of action, diffusion among selected populations of drug abusers as well as metabolism of novel psychoactive substances based on their detection in biological specimens, such as hair samples.

Further investigations to improve results obtained in qualitative screening should mainly focus on performance of semi-quantitative determination of detected drugs.

## Experimental

### Chemicals

Acetonitrile, methanol, ammonium formate (LC–MS grade) were purchased from Sigma-Aldrich (St. Louis, USA). Formic acid (FA) was purchased from Merck (Darmstadt, Germany). Acetone and hexane (analytical grade) were purchased from POCH (Gliwice, Poland). Sodium hydroxide, ethyl acetate, and hydrochloric acid (analytical grade) were obtained from POCH (Gliwice, Poland). Nylon (PA) ProFill™ 25 mm bright blue (0.2 μm pore size) syringe filters Whatman Puradisc™ 13 mm PTFE (0.2 μm pore size) syringe filters were purchased from Sigma-Aldrich (St. Louis, USA). Ultrapure H_2_O was prepared using HLP_5_ system from Hydrolab (Wiślina, Poland).

### Herbal products collection and preparation

The research collaborator collected four samples of legal high products available and sold on the drug market over the Internet under the names of “Tajfun”, “The recidivist”, “R.I.P.”, and “The Bandit”. For toxicological screening 50 mg of dried herbal material was used. Subsequently, 5 cm^3^ of solvent mixture ACN:MeOH (1:1) was added and the content was vortex mixed for 10 min. The mixture was sealed with aluminum for protection from light and left for 72 h in darkness. After incubation content was mixed for 2 min and consecutively filtered through syringe filters (0.2 μm pore size). Prior to analysis solution was diluted 1:100 with acetonitrile/water mixture (3:7). Subsequently it was transferred into autosampler vial. 10 mm^3^ were injected for Q-TOF-MS analysis.

### Hair sample collection and preparation

**Fig. 4:**
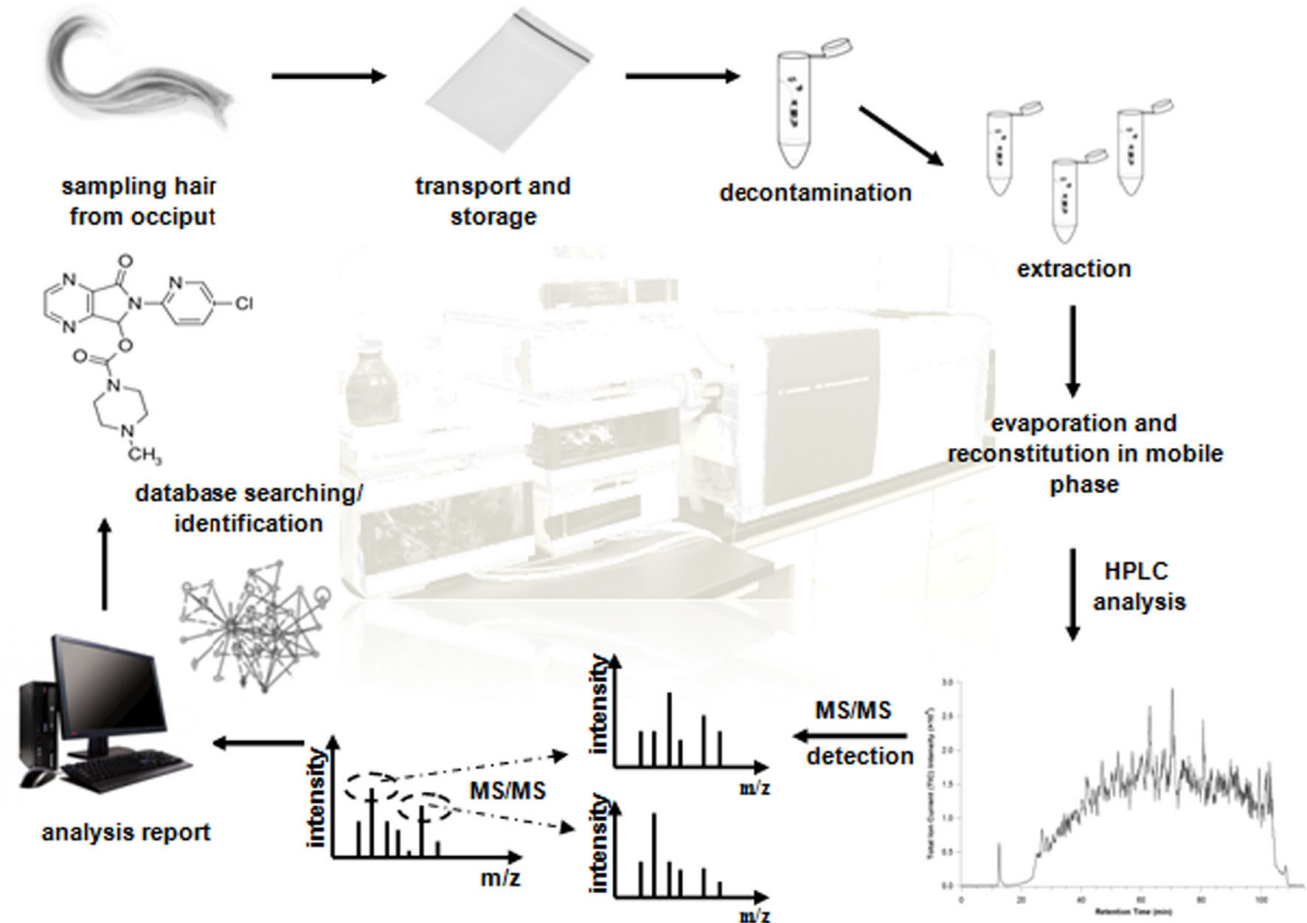
Workflow for identification of xenobiotics in hair sample

Hair samples (*n* = 13) were collected from the posterior vertex region of volunteers. Additionally, control hair samples were taken for analytical purposes from persons who do not declare drug intake to verify potential interferences coming from the hair matrix. The hair samples were stored in low-density polyethylene Zipper bags at 20–25 °C until the analysis. Before sample preparation hair sample (approx. 200 mg) was cut into small pieces. Then, the decontamination of the hair was performed in water bath by gentle shaking of hair for 1 min in 10 cm^3^ of *n*-hexane followed by shaking in 10 cm^3^ of acetone for 1 min. After hair drying on a filter paper (up to 10 min) samples were cut to 1–2 cm pieces and homogenized. In order to choose the most appropriate extraction method three procedures were compared: (1) 18 h incubation at 50 °C in MeOH in ultrasonic bath, (2) 8 h incubation at 50 °C in MeOH/ACN (1:1) in ultrasonic bath, and (3) 18 h incubation at 50 °C in 2.5 M NaOH. Evaluation of the most appropriate extraction method was done based on the relative comparison of the number of extracted substances from real hair samples or/and their chromatographic peak intensity using different extraction methods. Alkalic incubation was selected as optimal extraction method due to the fact, that it allows to extract the largest number of psychoactive substances relative to another tested methods from all investigated hair samples. It presumptively allowed to liberate contained xenobiotics. Optimized extraction conditions were as follows: after decontamination step, hair samples were incubated in 5 cm^3^ 2.5 M NaOH at 50 °C for 18 h and extracted in separator with 5 cm^3^ of ethyl acetate. After extraction, followed by centrifugation at 6000 rpm, organic phase was collected and the excess of solvent was evaporated under a gentle stream of nitrogen at 40 °C. The residue was reconstituted in a mixture of H_2_O and MeOH with 0.05 % FA (50:50, v/v) It is worth to mention that the colloid formation was recorded after 2 days of extract storage at room temperature. Finally, 10 mm^3^ of the extract was injected to perform HPLC-ESI-Q-TOF-MS analysis. Overall workflow for the identification of drugs and psychoactive substances in hair sample has been shown in the Fig. 4.

### Instrumentation

The HPLC-Q-TOF-MS was performed with the use of an Agilent 1290 LC system equipped with a binary pump, an online degasser, an autosampler and a thermostated column compartment coupled with a 6540 Q-TOF-MS with a Dual ESI source (Agilent Technologies, Santa Clara, CA, USA). An Agilent ZORBAX XDB-C-8, 150 × 4.6 mm, 3.5 μm column was used for RP-HPLC separation of extracts obtained from hair samples and herbal products with gradient elution program from 10 to 100 % B during 20 min followed by 100 % B maintained for 5 min. The mobile phase flow rate was 0.5 cm^3^/min and injection volume in this case was 10 mm^3^. The mobile phase consisted of water containing 0.01 % formic acid (component A) and methanol containing 0.01 % formic acid (component B). The column temperature throughout the separation process was kept at 40 °C. During all analyses, the samples were kept in an autosampler at 4 °C.

The ESI source was operated both in positive and negative ion mode with the following conditions: the fragmentor voltage was set at 120 V, nebulizer gas was set at 35 psig, capillary voltage was set at 3500 V, and drying gas flow rate and temperature were set at 10 dm^3^/min and 300 °C, respectively. For MS/MS measurements collision energy ramp ranging from 15 to 40 eV to promote fragmentation was used. The data were acquired in centroid and profile mode using High Resolution mode (4 GHz). The mass range was set at 50-1000 *m/z* in MS and MS/MS mode. The data were processed with the MassHunter Workstation QualitativeAnalysis.B.03.01 Software. The Q-TOF-MS was calibrated on a daily basis.
